# Auditory recognition of familiar and unfamiliar subjects with wind turbine noise

**DOI:** 10.3390/ijerph120404306

**Published:** 2015-04-17

**Authors:** Luigi Maffei, Massimiliano Masullo, Maria Di Gabriele, Nefta-Eleftheria P. Votsi, John D. Pantis, Vincenzo Paolo Senese

**Affiliations:** 1 Department of Architecture and industrial Design “L.Vanvitelli”, Seconda Università degli Studi di Napoli, Via San Lorenzo, 81031 Aversa (CE), Italy; E-Mails: luigi.maffei@unina2.it (L.M.); maria.digabriele@unina2.it (M.D.G.); 2 Department of Ecology, School of Biology, Aristotle University, U.P. Box 119, Thessaloniki postal code 54124, Greece; E-Mails: nvotsi@bio.auth.gr (N.-E.P.V.); pantis@bio.auth.gr (J.D.P.); 3 Department of Psychology, Seconda Università degli Studi di Napoli, Viale Ellittico 31, 81100 Caserta, Italy; E-Mail: vincenzopaolo.senese@unina2.it (V.P.S.)

**Keywords:** wind turbine noise, noise detection, familiar noise

## Abstract

Considering the wide growth of the wind turbine market over the last decade as well as their increasing power size, more and more potential conflicts have arisen in society due to the noise radiated by these plants. Our goal was to determine whether the annoyance caused by wind farms is related to aspects other than noise. To accomplish this, an auditory experiment on the recognition of wind turbine noise was conducted to people with long experience of wind turbine noise exposure and to people with no previous experience to this type of noise source. Our findings demonstrated that the trend of the auditory recognition is the same for the two examined groups, as far as the increase of the distance and the decrease of the values of sound equivalent levels and loudness are concerned. Significant differences between the two groups were observed as the distance increases. People with wind turbine noise experience showed a higher tendency to report false alarms than people without experience.

## 1. Introduction

Over the last few years climate change has triggered global concern leading to public awareness and policies regarding sustainable energy provision [1]. In 2007 the European Union adopted the “20-20-20” targets concerning a 20% reduction of greenhouse gas emission from 1990 levels, 20% increase in the consumption of energy produced from renewable resources and a 20% improvement in energy efficiency [2]. Under the framework of Renewable Energy [3], the European Commission established measures to provide 23.5% of the electricity and 12% of the overall primary consumption by means of renewable energy sources [4]. Nowadays the development of renewable energy installations is growing rapidly [5], resulting in a remarkable number of innovative applications of alternative energy sources [6]. Wind power development is one of the most technically advanced renewable energy technologies and thus wind farms, as electricity generators, are widely expanding in Europe [7], with an increasing number and size of wind turbines [8]. Wind turbine installations provide profitable investments, under an environmental-friendly concept [9].

From one point of view, it has been documented that wind power plants do not emit any chemical compounds dangerous to human health, while at the same time, they reduce the adverse effects of mining and transporting fossil fuels. They produce electricity in a cost-effective way, even in remote areas, contributing to their development. Moreover they are easy to construct, operate and maintain. Last but not least, wind farms can safeguard the autonomy, and consequently security of a country’s energy supplies with a sustainable perspective.

On the other hand, there are several negative effects of wind farms which can be attributed to noise and low frequency sound emission, the stroboscopic and the shadow flicker phenomena, the ice throw risk, the interference with radio and television signal and the electromagnetic field exposure [10]. Aside from the direct and obvious impacts of wind turbines, it has also been recorded that wind farms alter land use types, reduce biological activities, affect bird and bat ecology [4,11,12], change the microclimate conditions and interfere with the flow of surface and underground water [13,14]. Among these disadvantages of wind farms, the soundscape and the scenic quality degradation of the landscape [15,16,17,18] prevail in public complaints [19,20,21,22]. 

Furthermore, though wind farms have contributed to the reduction of CO_2_ emissions during the past 20 years [5], they also generate other types of emissions such as sonic ones. The sounds generated by wind turbines can be categorized as mechanical—caused by the interaction of turbine components, and aerodynamic‒due to the air flow over the blades [23,24]. They also depend on atmospheric factors and can be classified as tonal or broadband [23,24], low frequency [8,23,25], impulsive [23,26] and with amplitude modulation [27]. 

Researchers have found that other sound properties, different from the equivalent A-weighted SPL, influence the perception and annoyance arising from wind turbine noise [25,26,27]. Depending on these properties wind turbine noise was perceived as lapping, switching, whistling [26] or depending on the distance from the wind farm and the day period, as a thumping or rumpling noise [28].

In quiet environments, most frequently occurring conditions for the installation of these plants, wind turbine noise strongly characterizes their soundscape. It represents the noise most recognized by inhabitants and coexists with the blowing wind noise, causing a mutual masking [26], varying according to the different operating conditions of wind turbines [29].

For people living in the vicinity of wind farms, the perception of their environmental impact depends on several physical (e.g., acoustic, visual) [30] and individual (e.g., psychological, socio-economic, political) factors [31,32,33].

Studies [26,31,32,34] have shown that wind turbine noise is more annoying than other community noise sources with the same A-weighted sound level. Van Renterghem [35] observed that the wind turbine noise is not perceived so different from the highway one, when it is not known beforehand.

Special concern has been paid to the potential health effects of wind farms. Though it is suggested that, except from ear damage and general annoyance [34,36,37], wind turbine noise can be correlated to physical health problems, scientific proofs are still missing [8,34,38].

Nevertheless, the low frequency sound emitted by wind turbines could be associated to general annoyance and sleep disturbances [10,39], sometimes leading to psychological symptoms related to the autonomic nervous system, the neuroendocrine system, the immune system [37], and the cardiovascular system [40] or fatigue, headache, and impaired concentration [26,41], resulting in the so-called “Wind Turbine Syndrome” [42].

Chapman *et al*. [43] in a study involving 51 wind farms across Australia, showed large spatio-temporal differences in the distribution of wind farm noise and health complaints. They pointed out that in the vast majority of wind farms operated in Australia neither noise nor health complaints had been reported until 2009, when they began to be registered under the influence of anti-wind farm activism, stressing health problems. This seems consistent with the hypothesis that “wind turbine syndrome”, and the seemingly boundless range of symptoms associated with it, has important psychogenic nocebo dimensions.

Different researchers have identified other factors which influence noise annoyance of wind farms: negative oriented personality (NOP) traits [44], the attitude to wind turbines [26,45], and noise sensitivity [26]. Moreover it's worth mentioning that people who benefit economically from wind farms do not report any annoyance [32,39,43].

Despite the wide spectrum of aspects related to the wind turbine noise faced by previous studies, the difference between the percentages of annoyance due to wind turbine noise and other infrastructures was not sufficiently explained. Recently, van Renterghem *et al*. in a subjective test of the annoyance, detection and recognition of wind turbine noise [35], concluded that the awareness of the source is a relevant aspect for the noise perception and inter-individual differences could allow some people to detect and recognize wind turbine noise more easily, even if its presence is not revealed. 

As regards the auditory event perception, Gaver [46], in his ecological approach, has distinguished “everyday listening” from “musical listening”. According to his theoretical framework, in musical listening, people process the perceptual components of the auditory information (audio-oriented listening), whereas in everyday listening, people process the semantic components of the auditory information (source-oriented listening). In the latter case, the auditory information triggers the multidimensional memory trace associated to the stimulus. In this sense many evidences have shown the cross-modal integration between auditory and visual stimuli [47] while other studies support the idea that also the low frequencies and infrasound could play a role, when they are perceived as a vibratory sensation by the mechanoreception of the human trunk [48] and head [49] or by the skin receptors [50].

In parallel with the hypotheses of Gaver [46], Marcell *et al*. [51], utilizing a wide range of environmental sounds suggested that, when listening to environmental sounds, people are set with the everyday listening mode, more than the musical listening, whereas Gygi *et al*. [52], by comparing the perception of real and imagined sounds, suggested that for familiar sounds, the listeners’ auditory memory influences judgments made when the sound is actually present. Eventually, a recent study showed that people living close to wind turbines for long time are well aware of the presence of this noise source. Moreover the consequent daily exposure results in becoming more expert or sensitive to this type of noise [53].

In this paper an auditory experiment on the recognition of wind turbine noise for people with a prolonged exposure to this noise source is presented. Participants were presented some environmental sounds of wind turbines recorded at different distances and the relative control sounds (wind only). The auditory test was limited to the audible range. Two groups of participants were taken into consideration, a first group named familiar, was constituted by people that had lived at least 6 months close to wind turbines and do not have any economic benefits relative to the installation of the wind turbines. The second group, named unfamiliar or control, was composed by adults that have never been exposed to or experienced wind turbine noise. The two groups were matched in terms of noise sensitivity.

According to the ecological framework [46] and the recent evidences [53], we expected that when exposed to the turbine noise, familiar and control participants should show different patterns of responses. In other words, because of the prolonged previous experience, the familiar participants should process the stimuli by adopting an everyday listening mode, while the controls are expected to adopt a musical listening one. Therefore, the familiar listeners should be less able to distinguish the turbine noise from the environmental sound than the controls. The materials, the methodology and the results of the experiment are presented and discussed in the next paragraphs. 

## 2. Experimental Section 

### 2.1. Sound Recording and in situ Measurements 

**Figure 1:**
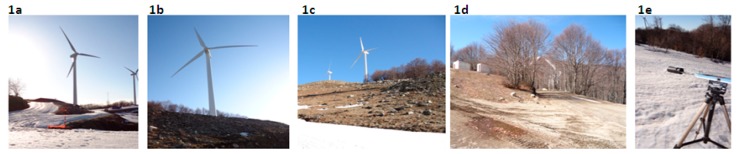
Pictures of the recording positions at: (**a**) 150 m; (**b**) 200 m; (**c**) 1500 m; (**d**) >2500 m. In (**e**) is shown the calibration of the sound level meter *in situ*.

Several sessions of recordings were performed at the wind farm of the Toumpa-Anthovouni region of the prefecture of Florina in Greece. The wind farm is composed of 34 wind turbines, each one of a power of 0.85 MW and a hub height of 50 m. Sound recordings of about 5 min, were made at five distances (150, 200, 250, 300 and 1500 m, Figure 1a–d) from the closest wind turbine. Furthermore, an additional sound recording was conducted at a distance greater than 2500 m from the nearest wind turbine. This position was chosen so as to be far enough away that the wind turbine noise was no longer audible and, in this research, is named wind-only distance or condition. All the recordings were made during daytime, when the wind turbines were functioning at about 20 rpm, the temperature of the ground height was about 11 °C and the sky was clear. During the measurements the wind turbine noise was the main sound source of the soundscape and the background noise was represented exclusively by the interaction between the wind and the surrounding vegetation. The sound recordings were made by means a Tascam DA-P1-DAT portable recorder and a Schoeps MS microphone system. Moreover, to measure the sound equivalent level of each sound recording a sound level meter (Cesva SC-310), calibrated with a 1 kHz signal of 94 dB generated by a calibrator (model CB006) (Figure 1e), was positioned close to the microphone.

### 2.2. Sound Stimuli 

For each measurement condition (D_150_, D_200_, D_250_, D_300_, D_1500_, D_wind_only_), five representative sound tracks of 9 s were selected to be used as auditory stimuli in following test. Each soundtrack was further characterized acoustically in terms of sound equivalent levels, L_eq,A_ and of the average values of the main psychoacoustics indexes: Loudness (N), Fluctuation Strength (F), Sharpness (S), Tonality (T), Roughness (R). The analyses of the data were carried out by means of the software dBFA. 

The data in Table 1 show how, for the recordings at distances lower or equal than 250 m, the sound equivalent levels, L_eq,A_, were strongly influenced by the wind turbine noise, on average between 35.3 and 38.5 dB(A), while at greater distances, the L_eq,A_ were lower and quite stable, on average in the 28.8 to 30.7 dB(A) range. The higher sound levels at 200 m, as well as the smaller sound levels fluctuations at different distances, might be attributed to several factors. The most important are the changes of wind speed and direction as well as the choice of the recording position within the WTs’ directivity pattern. 

**Table 1:** Sound equivalent levels, in dB(A), for the 5 soundtracks at the six distances.

Soundtrack No.	Distance					
	D_150_	D_200_	D_250_	D_300_	D_1500_	D_wind_only_
1	36.5	39.1	35.7	30.1	28.6	31.1
2	36.3	38.4	35.9	29.2	28.5	30.7
3	34.5	39.0	35.7	29.2	28.9	29.2
4	34.5	38.5	35.8	29.5	28.6	31.2
5	34.5	37.7	35.8	29.9	29.2	31.3
Average	35.3	38.5	35.8	29.6	28.8	30.7

A further analysis of the low frequency content showed that all the differences between dB(C) and dB(A) of the stimuli were in the range 3–4 dB.

The analyses of the average values of psychoacoustic indexes, in Table 2, show that: the Tonality was greater than zero only at the distance of 150 m, while the Fluctuation Strength at distances lower than 300 m, with the maximum value at 200 m. The Sharpness and Roughness variation were quite weak in relation to distance. As expected the values of the loudness (N) were found to have the same behaviour observed for the sound equivalence levels. 

**Table 2:** Psychoacoustic indexes. Mean values at the six distances.

Index	Unit	Distance					
		D_150_	D_200_	D_250_	D_300_	D_1500_	D_wind_only_
Loudness (N)	sone	2.314	3.030	2.284	1.226	1.154	1.538
Fluctuation Strength (F)	vacil	0.008	0.030	0.022	0.000	0.000	0.000
Sharpness (S)	acum	0.960	0.970	0.874	0.804	0.884	0.882
Tonality (T)	tu	0.004	0.000	0.000	0.000	0.000	0.000
Roughness (R)	asper	6.756	6.710	6.654	6.490	6.234	6.310

For each sound track the FFT of the sound signals were extracted and averaged. Then, according to the procedure [54] the audibility at low frequency of the selected sound tracks was verified. For the application of this procedure the three following Equations were used, in the respective range of frequency:

(1) $${\text{Att}}_{\text{(2-20Hz)}}\text{= }-\text{1.0183}\cdot {\text{10}}^{\text{-2}}\cdot {\text{f}}^{\text{3}}\text{ + 3.8537}\cdot {\text{10}}^{\text{-1}}\cdot {\text{f}}^{\text{2}}-\text{ 6.3935}\cdot \text{f + 133.48 }\left[\text{dB}\right]$$

(2) $$\begin{array}{l} {\text{Att}}_{\text{(20-200Hz)}}=1.5948\cdot 10^{-11}\cdot f^{6}-1.3537\cdot 10^{-8}\cdot f^{5}+4.5945\cdot 10^{-6}\cdot f^{4}-8.0269\cdot 10^{-4}\cdot f^{3}+\\ 7.7761\cdot 10^{-2}\cdot f^{2}-4.2624\cdot f\text{+}137.99\left[\text{dB}\right] \end{array}$$

(3) $${\text{Att}}_{\text{(200-500Hz)}}=-1.3635\cdot 10^{-7}\cdot f^{3}+2.2850\cdot 10^{-4}\cdot f^{2}-1.399\cdot 10^{-1}\cdot f\text{+}34.306\left[\text{dB}\right]$$

**Figure 2:**
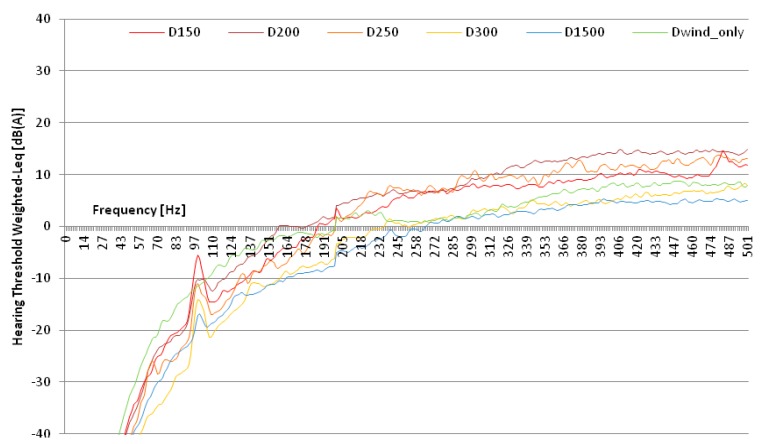
FFT of the hearing threshold weighted-Leq for the sound tracks groups D_150_, D_200_, D_250_, D_300_, D_1500_ and D_wind_only_.

The analysis shows that, for all groups, the sound levels at low frequency were higher than the hearing threshold (Figure 2) only from 190 Hz.

## 3. Methodology

### 3.1. Test Set-Up 

The 30 selected sound tracks (5 tracks × 6 distances) constituted the audio stimuli for the subjective auditory test. A Two-Alternative Forced Choice (2-AFC) auditory test was prepared and administered using the software Psychopy [55]. Participants could read the questions on the screen and listen to the sound stimuli through a supra-aural headphone connected to the same laptop. 

According to ethical principles, the participants were preliminarily informed about the general context of the anonymous test without being totally enlightened about its purpose. Participants were also aware of test’s duration and the procedure, as well as of the right to decline or withdraw from participating in the test. Before starting the test, participants were asked if they had significant hearing problems in their lifetime. The test procedure was divided into different phases. At the beginning of the test, participants had to fill out a questionnaire regarding: age, gender, qualification, occupation, municipality of residence, dwelling area, marital status and partner’s qualification and occupation. Next they were asked: (a) if they lived close to wind farm; in case of affirmative answer, how long they have lived close to the wind farm, and (b) if they support wind turbine installation. 

Furthermore, in order to evaluate the subjective noise sensitivity, they were administered the Weinstein’s noise sensitivity scale [56]. For every statement, participants had to indicate their agreement by means of a 6-point Likert scale, which ranges from “disagree strongly” to “agree strongly”.

Subsequently, the audio test started. After a brief introduction, during which the experimenter explained the instructions on the screen (10 s), a training session with seven audio stimuli was undertaken to familiarize participants with the procedure. The training session lasted about 2 min and 30 s. Afterwards, a sequence of 30 audio stimuli in a balanced order was presented to the participants.

For each audio stimulus the following procedure was followed:

(a) through a message on the screen (6 s), the subjects were asked to listen carefully to the sound stimulus played back by headphones;
(b) audio stimulus playback (10 s);
(c) the subjects were asked if the wind turbine noise was identified through the following question: “Have you identified the wind turbine noise in the last audio track? Please, answer pressing “y” (if yes) or “n” (if not)” on the keyboard (5 s). The whole procedure for each participant was completed in about 13 min.

### 3.2. Participants

A total of 40 participants, divided in two groups of 20 subjects, were involved in the audio test. Whereas the two groups had different experience as far as wind turbine noise exposure is concerned, they were found similar regarding some other characteristics The first group (familiar) was composed by 20 subjects, 12 male and eight female (mean age = 37.0; st. dev. 12.5). The participants of this group had lived close (within 800 m) to the wind farm for more than 6 months (nine for more than one year; seven since wind farm installation, about 4 years prior) and were exposed to the same wind turbine noise of the recorded sound stimuli. None of the participants had received any economic benefits relative to the installation of the wind turbines.

The second group (unfamiliar or controls) was composed by 20 subjects, nine males and 11 females (mean age = 38.9; st. dev. 13.8), never exposed to wind turbine noise in their lifetime. The participants of this group were matched to the participants of the familiar group as function of the noise sensitivity.

The 40 participants all reported to have normal hearing and no relevant hearing problems during their lifetime. All lived in rural areas and did not receive incentives to participate to the test. The test took place in their houses during a visit to the vicinity.

### 3.3. Data Analysis

To investigate if the effect of the prolonged exposure to the wind farm on the noise detection is moderated by the distance from the noise source, a 2 × 6 mixed ANOVA that treated the Group (familiars and controls) as a 2-level between-subject factor, the Distance as a 6-level within-subject factor (D_150_, D_200_, D_250_, D_300_, D_1500_ and D_wind_only_), and the detection ratings as dependent variable was performed. The Bonferroni correction was used to analyze *post hoc* effects, and the magnitude of the significant effects was indicated by partial eta squared (η^2^_p_). Moreover, to test if the effects of the Group and the Distance were independent from the noise sensitivity, the analysis was replicated by considering the WNSS score as a covariate (ANCOVA).

To take into account the false recognition responses and to get a unique discriminant score, the d’ scores were calculated as a function of the distance. The d’ scores were computed by subtracting the z-scores of false recognition associated with the wind-only condition from the z-scores of the correct recognitions associated to each condition [57]. Therefore a 2 × 5 mixed ANOVA, that treated the Group (familiars and controls) as a 2-level between-subject factor, the Distance as a 5-level within-subject factor (D_150_, D_200_, D_250_, D_300_ and D_1500_) and d’ score as dependent variable, was performed.

Eventually, to investigate the association between the sound equivalent levels, the loudness and the noise detection responses, ratings were averaged over participants as a function of the condition and the correlation coefficients were computed.

## 4. Results

**Figure 3:**
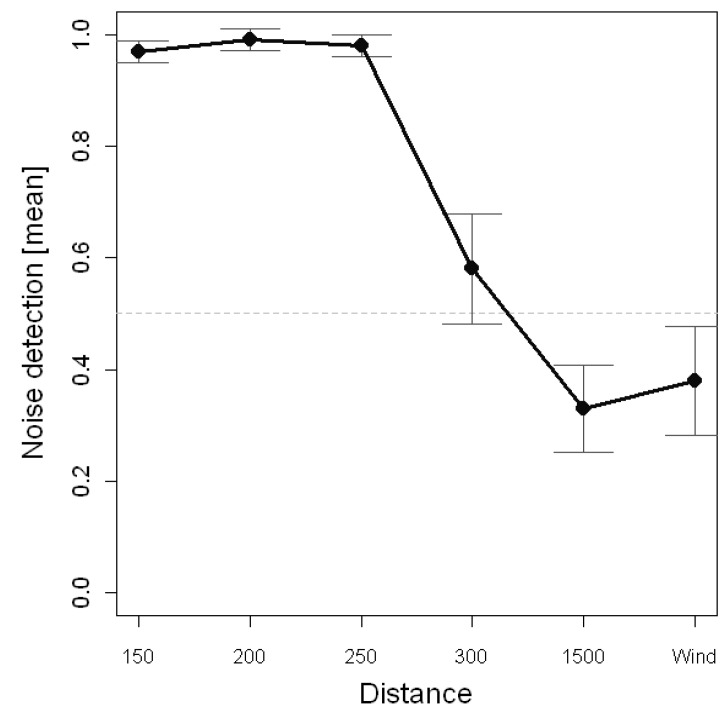
Noise detection ratings as a function of distance.

The ANOVA on the noise detection ratings showed that the capacity to discriminate the wind farm noise was influenced by the Group, *F*(1, 38) = 4.421, *p* = 0.042, *η*^2^_p_ = 0.104, the Distance, *F*(5190) = 108.337, *p* < 0.001, *η*^2^_p_ = 0.740, and the Group × Distance interaction, *F*(5190) = 3.869, *p* < 0.01, *η*^2^_p_ = 0.092. The mean comparison revealed that participants of the familiar group (*M* = 0.75) were more prone to identify the wind farm noise than participants of the control group (*M* = 0.66). The *post hoc* analyses for the Distance effect revealed that the conditions affected differently the proportion of noise detection (Figure 3). In particular, participants were more prone to identify the wind farm noise when the sound was registered at 150, 200 or 250 m (*M* = 0.97, *M* = 0.99 and *M* = 0.98, respectively) than under all other conditions. The lowest tendency to recognize the presence of the wind turbine noise was observed at 1500 m (*M* = 0.33). The wind-only condition showed that participant had a tendency to rise false alarms (*M* = 0.38) with a rating similar to the 1500 m one.

**Figure 4:**
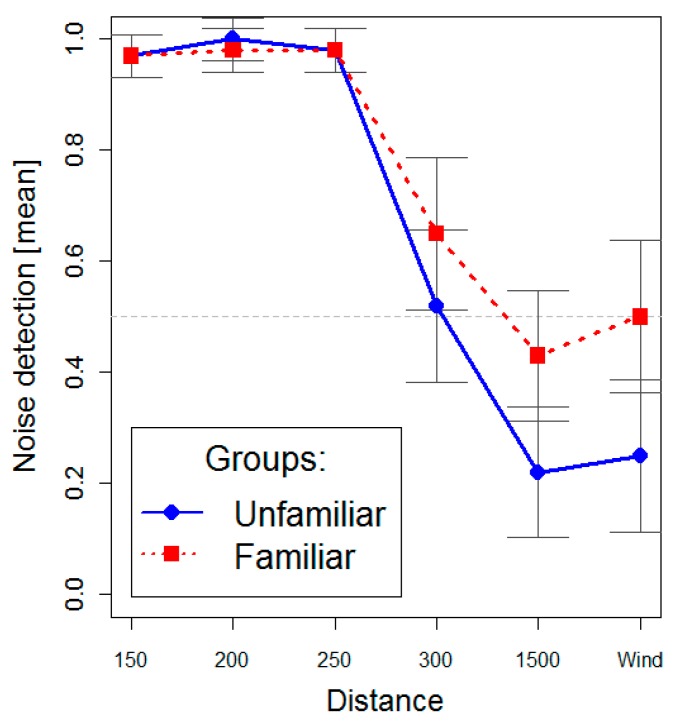
Noise detection ratings as a function of group and distance.

The *post hoc* analyses for the Group × Distance interaction revealed that no significant differences were observed between the familiar and the control group for the 150, 200, 250 and 300 m distances, and that the differences between the two groups were observed only at 1,500 m and in the wind-only conditions (Figure 4).

The ANCOVA that considered the noise sensitivity as covariate confirmed the same significant effects.

The *ANOVA* on the d’ score confirmed that participants were more able to better identify the presence of wind turbine noise at the distances of 150 m, 200 m and 250 m (*M* = 3.48; *M* = 3.79 and *M* = 3.72, respectively) than at both 300 m (*M* = 1.22, *p* < 0.05) and 1,500 m (*M* = −0.10, *p* < 0.05), and that the latter two conditions were significantly different from each other (*p* < 0.05). Moreover, the results highlighted that the familiar group was more prone to report false alarms (*M* = 1.91) than the control group (*M* = 2.93).

The correlation analyses showed that noise detection ratings were positively associated with the sound equivalent levels and the loudness. In both groups, the higher the sound equivalent level was, the higher the noise detection rating scored, *r* = 0.819 and *r* = 0.873, *p*_s_ < 0.001, *N* = 30, respectively for the familiar and the control groups; and the higher was the Loudness the higher was the noise detection rating, *r* = 0.782 and *r* = 0.839, *p*_s_ < 0.001, *N* = 30, respectively for the familiar and the control groups.

## 5. Conclusions

The results of these subjective experiments are consistent with the numerical data of the stimuli. As expected, for both groups of participants, familiar and unfamiliar, the trend of the auditory recognition is congruent with the increase of the distance and the decrease of the values of sound equivalent levels and loudness. 

To better understand the complexity of the auditory recognition test results, three different regions were identified: (1) a Proximity Zone (from 150 to 250 m); (2) a Transition Zone (300 m) and (3) a Far Zone (1500 m). In the Proximity Zone the wind turbine noise is clearly distinguishable; in the Transition Zone the wind turbine noise becomes weaker, while in the Far Zone the contribution of the wind turbine on the environmental noise can be considered negligible or null. In this latter region the main sound is due to the blowing wind and its interaction with the surrounding vegetation. 

As regards the differences between familiar and unfamiliar subjects, the results of the recognition rate showed that no significant differences were detected in the Proximity Zone; that the difference between the two groups became significant in the Transition Zone; and that the difference increased as the distance from the wind turbines increased (Far Zone). Moreover, as illustrated by the recognition rate associated to the wind-only tracks, data showed that the familiar listeners had a higher tendency to report false alarms than the controls.

The analyses on the d’ score confirmed the same pattern as the results, that is, participants were more able to identify the presence of wind turbine noise in the Proximity Zone than in the Transition zone or the Far zone, and that the familiar listeners were more prone to report false alarms in the wind turbine noise detection than the controls.

In line with the relevant literature [46,51,52,53], the differences in the Transition Zone and in the Far Zone confirm that when wind turbine noise is less distinctive, the subjective experience becomes more relevant to the recognition of wind turbine noise. In this condition, participants with a previous experience of wind turbine noise are mainly focused on the sound-producing event and its environment, more than on the perceptual dimensions or the physical attributes of the sound itself. Therefore, a possible explanation for the rate of false positive recognition could be that, in the familiar group, the exposures to familiar auditory stimuli automatically re-activate the multisensory memory trace associated to the wind turbine noise and the environmental context in which they are usually listened. According to our expectations, data showed that the percentage of wind turbine noise recognition for familiar subjects in an environmental sound with a windy zone is comparable to the recognition of unfamiliar subjects at the distance of 300 m.

The main limitation of this study is that the test was limited to the audible frequency range. This implies that, even though the low frequency contributions of the sound stimuli were very low, L(C) − L(A) < 4 dB, it is not possible to exclude that during the tests of the familiar subjects they were able to perceive, by the whole body, additional vibratory stimulus due to low frequencies or infrasound. 

Future research should draw attention to the zones where the subjective perception of wind turbines noise is strongly influenced by the experience of noise exposure, that is, the Transition Zone and the Far Zone. Moreover, laboratory experiment involving familiar and unfamiliar subjects could exclude possible cross-modal interaction due to low frequency and infrasound. Last, but not least, more extensive auditory tests with sound stimuli of wind-only conditions should be carried out, to better investigate the reactions of individuals exposed to turbine noise as far as the perception of wind sound is concerned. These latter researches could help to clarify the mechanisms that determine the high annoyance related to wind turbine noise.
